# Miniemulsion RAFT Copolymerization of MMA with Acrylic Acid and Methacrylic Acid and Bioconjugation with BSA

**DOI:** 10.3390/nano9060828

**Published:** 2019-05-31

**Authors:** Débora Vieira Way, Rayany Stôcco Braido, Sabrina Alves dos Reis, Flávio Alves Lara, José Carlos Pinto

**Affiliations:** 1 Programa de Engenharia Química/COPPE-Universidade Federal do Rio de Janeiro, Cidade Universitária 68502, Rio de Janeiro 21941-972 RJ, Brazil; dway@peq.coppe.ufrj.br (D.V.W.); rayany.sb@hotmail.com (R.S.B.); 2 Laboratory of Cellular Microbiology, Oswaldo Cruz Institute, Oswaldo Cruz Foundation, Rio de Janeiro 21045-900RJ, Brazil; sabrina.alvesreis@yahoo.com.br (S.A.d.R.); flavioalveslara2000@gmail.com (F.A.L.)

**Keywords:** RAFT, bioconjugation, copolymers, maleimide-PEG-NHS

## Abstract

Polymerization through reversible addition-fragmentation chain-transfer (RAFT) polymerization has been extensively employed for the production of polymers with controlled molar mass, complex architectures and copolymer composition distributions intended for biomedical and pharmaceutical applications. In the present work, RAFT miniemulsion copolymerizations of methyl methacrylate with acrylic acid and methacrylic acid were conducted to prepare hydrophilic polymer nanoparticles and compare cell uptake results after bioconjugation with bovine serum albumin (BSA), used as a model biomolecule. Obtained results indicate that the RAFT agent 2-cyano-propyl-dithiobenzoate allowed for successful free radical controlled methyl methacrylate copolymerizations and performed better when methacrylic acid was used as comonomer. Results also indicate that poly(methyl methacrylate-co-methacrylic acid) nanoparticles prepared by RAFT copolymerization and bioconjugated with BSA were exceptionally well accepted by cells, when compared to the other produced polymer nanoparticles because cellular uptake levels were much higher for particles prepared in presence of methacrylic acid, which can probably be associated to its high hydrophilicity.

## 1. Introduction

Polymerization through reversible addition-fragmentation chain-transfer (RAFT) is a type of radical polymerization that allows for the production of macromolecules with complex and controlled molecular architectures, including block, graft and star structures, with narrow molar mass distributions [1,2]. The use of the RAFT agent in particular leads to the production of polymers with narrow molar mass distributions because the reaction mechanism includes constant activation and deactivation of the polymer chains [1,2,3]. These types of polymers are useful for pharmaceutical and biomedical applications, as it is desirable that materials employed in living bodies present controlled and predictable molecular structures, besides the obvious requirements of good biocompatibility and non-toxicity [2,3,4,5]. Narrow molar mass distributions and high hydrophilicity are also desirable in most biomedical applications to grant the polymer the ability to remain in the blood stream for sufficiently long periods of time and to assure safer, more predictable and more consistent outcomes [4,5,6]. Besides, the RAFT agent can also provide the functional groups that are necessary for posterior particle functionalization.

RAFT polymerizations can be conducted in bulk, solution, suspension, emulsion or miniemulsion processes [7,8]. However, it is not possible to guarantee that the performances of RAFT agents will be similar in different reacting media, as RAFT agents can be very sensitive to modification of the chemical composition of the reacting system. For this reason, the appropriate selection of the RAFT agent may not be trivial and certainly depends on the considered comonomer mixture [2,9]. To help the selection of the most appropriate RAFT agent for different reaction systems, design tables have been built and are available in the literature [2,10], although the performances of RAFT agents have not been analyzed and reported in the literature for most comonomer systems.

Solution RAFT copolymerizations of methyl methacrylate (MMA) and acrylic acid (AA) [11] and of MMA and methacrylic acid (MA) have already been performed successfully using dimethylformamide as solvent and cumyl phenyldithioacetate or 2-cyano-2-propyl dithiobenzoate as RAFT agents [12,13,14]. However, to allow for biomedical and pharmaceutical applications, polymer nanoparticles should be preferably obtained [15]. To produce polymer nanoparticles, RAFT polymerizations might be conducted in miniemulsion [16], but apparently RAFT copolymerizations of MMA and AA and of MMA and MA have not been performed yet in heterogeneous media. Consequently, it cannot be assured that RAFT miniemulsion copolymerizations of MMA and AA and of MMA and MA can be performed successfully, as observed in organic solutions.

In the present work, 2-cyano-2-propyl-benzodithioate (CPBD) was used as the suitable RAFT agent to perform miniemulsion copolymerizations of MMA and AA and of MMA and MA and to produce polymer nanoparticles with different degrees of hydrophilicity. The selection of CPBD was based on the fact that CPBD is the RAFT agent used most commonly in heterogeneous MMA polymerizations and copolymerizations [10,17], although to the best of our knowledge the use of CPBD in the proposed miniemulsion reaction systems has not been reported previously.

Polymers manufactured through RAFT polymerizations usually contain a thiocarbonylthio group placed at the chain end due to the characteristic chain transfer mechanism of RAFT reactions [1]. These thiocarbonylthio groups can be used for posterior functionalization of the polymer chains, including bonding to biomolecules [8,18].

Bioconjugation can be defined as the bonding of a biomolecule to another molecule, such as a polymer chain [19]. When a biomolecule is linked to a polymer chain, the resulting hybrid product can be used for development of targeted drug delivery systems, as the biomolecule can function as a biochemical signal for recognition of specific target cells [20]. Bovine serum albumin (BSA) is a protein largely used in bioconjugation studies and can also be used in targeted release systems [21,22]. For this reason, in the present work, BSA is used as a model biomolecule for bioconjugation with the produced RAFT polymers. The main goal pursued here was to investigate whether the different degrees of hydrophilicity of the produced polymer nanoparticles can affect the efficiency of the cell uptake.

**Figure 1:**
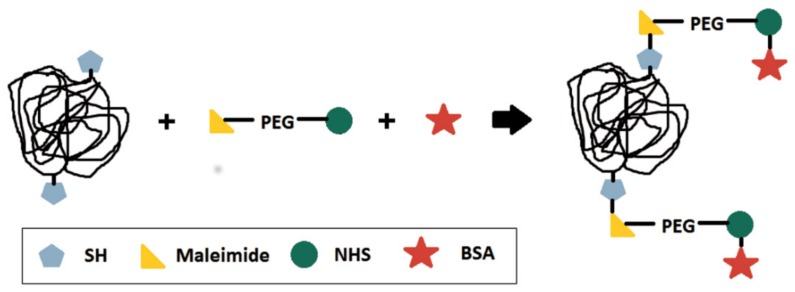
Scheme of the multistep bioconjugation reactions performed in the present work [10,23,24].

Bioconjugation of polymer nanoparticles with BSA is possible by using a maleimide-poly(ethylene glycol)-N-hydroxysuccinimide ester molecule as a spacer (or binder) [23]. To do that, the thiocarbonylthio groups present in the RAFT copolymers must be reduced to free thiol groups prior to the bioconjugation reaction [18,24]. The bioconjugation procedure is illustrated in Figure 1 to facilitate the comprehension of the proposed reaction scheme.

The reaction between maleimide and the free thiol groups can be classified as a thiol-ene click chemistry reaction, which means that the reaction is fast, can be performed under mild conditions and results in high yields [25,26]. Therefore, maleimide can be easily linked to the free thiol groups obtained after reduction of the thiocarbonylthio end-groups present in the RAFT (co)polymers [18,23]. Meanwhile, N-hydroxysuccinimide activated groups can also be linked to the free amine groups present in most biomolecules, such as BSA, for example [23,27].

Based on the previous paragraphs, the main objective of the present work was to produce polymer nanoparticles through RAFT miniemulsion copolymerizations of MMA and AA and of MMA and MA, using CPBD as the RAFT agent, and afterwards perform the bioconjugation of the produced nanoparticles with BSA, using maleimide-poly(ethylene glycol)-N-hydroxysuccinimide as a spacer. Finally, uptakes of polymer nanoparticles by cells were characterized and compared to each other.

## 2. Materials and Methods

### 2.1. Materials

Methyl methacrylate (MMA, with minimum purity of 99.5 wt %), acrylic acid (AA, with minimum purity of 99 wt %), hexadecane (HXD, with minimum purity of 99 wt %), sodium dodecyl sulfate (SDS, with minimum purity of 99 wt % and containing 10 wt % of water), ethylenediamine tetraacetic acid (EDTA, with minimum purity of 99 wt %) and dibasic potassium phosphate (with minimum purity of 98 wt %) were purchased from VETEC (Rio de Janeiro, Brazil). Methacrylic acid (MA, with minimum purity of 99 wt %) was supplied by Industria Quimica Taubaté (São Paulo, Brazil). L-cysteine (Cys, with minimum purity of 97 wt %), 2-cyano-2-propyl-benzodithioate (with minimum purity of 97 wt %) and rhodamine B isothiocyanate (provided as a mixture of isomers with minimum purity of 70 wt %) were purchased from Sigma-Aldrich (Rio de Janeiro, Brazil). Azobisisobutyronitrile (AIBN) was obtained from Akzo Nobel (Amersfoort, The Netherlands) with minimum purity of 99 wt %. Dimethyl sulfoxyde (DMSO) was obtained from Nuclear (Rio de Janeiro, Brazil), with minimum purity of 99 wt %. Deuterated chloroform was purchased from Cambridge Isotope Laboratories (Tewksbury, MA, USA) with minimum purity of 99 wt %. Sodium bicarbonate was purchased from Proquimios (Rio de Janeiro, Brazil) with minimum purity of 99.7 wt %. Hydroquinone and tetrahydrofuran (THF) (both with minimum purity of 99 wt %) were purchased from Tedia (Rio de Janeiro, Brazil). Poly(ethylene glycol)-N-succinimidyl ester (maleimide-PEG-NHS, with minimum purity of 99 wt %), with weight-average molecular weight (Mw) of 5000 Da, was purchased from NanoCS (Cheshire, United Kingdom). Unless stated otherwise, all chemicals were used as received.

### 2.2. Reactions

#### 2.2.1. RAFT Polymerization

The RAFT polymerization procedure used in the present work was based on a previous report [28]. An aqueous solution containing 80 g of distilled water, 1 g (3.48 × 10^−3^ mol) of SDS and 0.1 g (1.19 × 10^−3^ mol) of sodium bicarbonate was prepared and stored for later use. Simultaneously, an organic solution containing 19.11 g (1.91 × 10^−1^ mol) of MMA, 0.0784 g (4.77 × 10^−4^ mol) of AIBN, 0.6 g (2.65 × 10^−3^ mol) of hexadecane and 0.2144 g (9.69 × 10^−4^ mol) of 2-cyano-2-propyl-benzodithioate (CPBD) was prepared. When copolymerization was performed, 15 wt % (15% mol) of the MMA was replaced by either AA or MA. After complete solubilization of the chemical constituents in both organic and aqueous solutions, the aqueous phase was transferred to the flask that contained the organic solution and the mixture was stirred for 15 min at 500 rpm to form the pre-emulsion. The pre-emulsion was then submitted to sonication at 70 W (corresponding to 70% of the maximum power of a Branson sonifier, model 450D, Danbury, CT, USA) for 10 min. During sonication, the emulsion was kept inside an ice bath under continuous magnetic stirring of 500 rpm. The emulsion was then transferred to a round bottom flask and sealed with a rubber cap. Then, two needles were inserted into the rubber cap: one needle was attached to a nitrogen line to allow for inertization of the flask (through slow bubbling of nitrogen for 1 h), while the other needle was used as the outlet gas line to keep the pressure constant inside the flask. The system was kept inside an ice bath under magnetic stirring during the inertization procedure. After that, the flask was transferred to an oil bath kept at 80 °C, where the polymerization reaction was performed for 5 h under magnetic stirring of 500 rpm. Aliquots were withdrawn during the reaction to characterize the evolution of monomer conversion (through gravimetric analyses), particle size distribution (through dynamic light scattering) and molar mass distribution (through gel permeation chromatography) of polymer samples. The final products were not purified and were used as obtained in the following characterization and reaction steps.

#### 2.2.2. Bioconjugation

To ensure the reduction of the thiocarbonylthio groups, 500 mg of polymer latex, 5 mL of distilled water and 0.189 g (5.00 × 10^−3^ mol) of sodium borohydride were added to a 50 mL flask, covered with Parafilm and kept in a shaker at 25 °C and 100 rpm for 2 h. After that, 85 μL of concentrated hydrochloric acid and 0.087 g (4.99 × 10^−4^) of dibasic potassium phosphate were added and pH was adjusted to 5.5 with either HCl (1 M) or NaOH (40 wt %). After reduction of the thiocarbonylthio groups, an aqueous solution with pH of 5.5 was prepared, containing 10 μg/μL of maleimide-PEG-NHS Mw 5000. Then, 30 μL of this solution was added to 50 μL of the reduced latex in an Eppendorf. The Eppendorf containing the mixture was kept in a shaker at 25 °C and 100 rpm for 30 min. Finally, 35 μL of an aqueous solution containing 10 mg/mL of BSA were added to the mixture and incubated in a shaker at 37 °C and 100 rpm for 24 h. Finally, all samples were centrifuged at 8000 rpm for 10 min at 15 °C and washed three times with 100 μL of distilled water. It is important to highlight that the polymer latex was not purified before the bioconjugation procedure described above.

#### 2.2.3. Labeling with Rodamine B

First, 100 μL of 0.1 M sodium bicarbonate pH 9.0 buffer and 75 μL of a 10 μg/μL solution of Rodamine B in DMSO were added to the bioconjugated polymer. The mixture was kept in a shaker for 1 h at 100 rpm and 25 °C and then centrifuged at 8000 rpm for 10 min at 15 °C and washed 3 times with 100 μL of 0.1 M sodium bicarbonate pH 9.0 buffer.

### 2.3. Characterization

#### 2.3.1. Gel Permeation Chromatography (GPC)

GPC analyses were performed at 40 °C with a Viscotek VE2001 chromatograph (Malvern, Worcestershire, United Kingdom) equipped with a Viscotek VE3580 refractometric detector (Malvern, Worcestershire, United Kingdom), a Viscotek 2500 UV detector (Malvern, Worcestershire, United Kingdom), a Shodex KF-G pre-column (Showa Denko K.K., Tokyo, Japan), two Shodex KF-804 columns (Showa Denko K.K., Tokyo, Japan) and one Shodex KF-805 column (Showa Denko K.K., Tokyo, Japan). Calibration was performed with polystyrene standards with molecular weights ranging from 376 to 1.0 × 10^6^ Da. Samples were prepared with concentration of 1 mg/mL in THF. Solutions were filtrated with a Teflon filter with pore size of 0.45 μm. The injection volume was equal to 200 μL and the operating flow rate was equal to 1 mL/min. 

#### 2.3.2. Dynamic Light Scattering (DLS)

Particle size distributions were determined with help of a Zetasizer Nano ZS (Malvern, United Kingdom) equipped with a 4 mW 632.8 nm laser. Samples were prepared by diluting 1 droplet of the produced polymer latex with distilled water until the maximum level of a glass cuvette used to perform the analyses. All measurements were conducted at 25 °C.

#### 2.3.3. Nuclear Magnetic Resonance (NMR)

^1^H NMR spectra were recorded at frequencies of 400 MHz using a Bruker Avance III spectrometer (Bruker, Santa Barbara, CA, USA) equipped with a 5 mm probe and set to 25 °C. Samples were prepared by diluting 15 mg of each previously dried polymer sample in 0.8 mL of CDCl_3_.

#### 2.3.4. Contact Angle

Contact angle measurements were conducted in a Dataphysics Goniometer (model OCA 20, Dataphysics, Filderstadt, Germany). A small latex portion was dried in a recirculation oven at ambient temperature and grinded with help of mortar and pestle. Then, a small portion of the obtained powder was placed in a microscope slide and manually compressed with another slide until obtainment of a completely smooth surface. Finally, a drop of distilled water was gently deposited on the surface of the polymer film for contact angle measurements.

#### 2.3.5. Bioconjugation Efficiency

The quantification of the conjugated BSA was performed with help of the well-known Bradford methodology [29]. The results were monitored by light absorption at 595 nm with help of a UV-Vis spectrophotometer (Perkin Elmer, model Lambda 35, Norwalk, CT, USA).

#### 2.3.6. Cellular Uptake

To observe the uptake of conjugated beads by cells, mouse macrophage (RAW 267.4) cells, cultivated in a Dulbecco’s Modified Eagle Medium–high glucose (LGC, São Paulo, Brazil) supplemented with 10 vol % of fetal bovine serum, were exposed to the bioconjugated polymer beads labeled with Rhodamine B. After 3 h at 37 °C, cells were washed three times with phosphate-buffered saline (PBS) solution and submitted to detachment. Detachment was performed with help of cell scrapers and a cytometry buffer (10 vol % fetal bovine serum in PBS). Cell suspensions were then placed on ice bath and immediately subjected to the acquisition of 100,000 events with help of a FACS Calibur system (Becton & Dickinson, Franklin Lakes, NJ, USA), using the FL3 fluorescence channel. Cells that were not exposed to the bioconjugated polymer beads were assumed to provide the baseline 0% signal and used for validation of the experimental procedure.

## 3. Results

**Figure 2:**
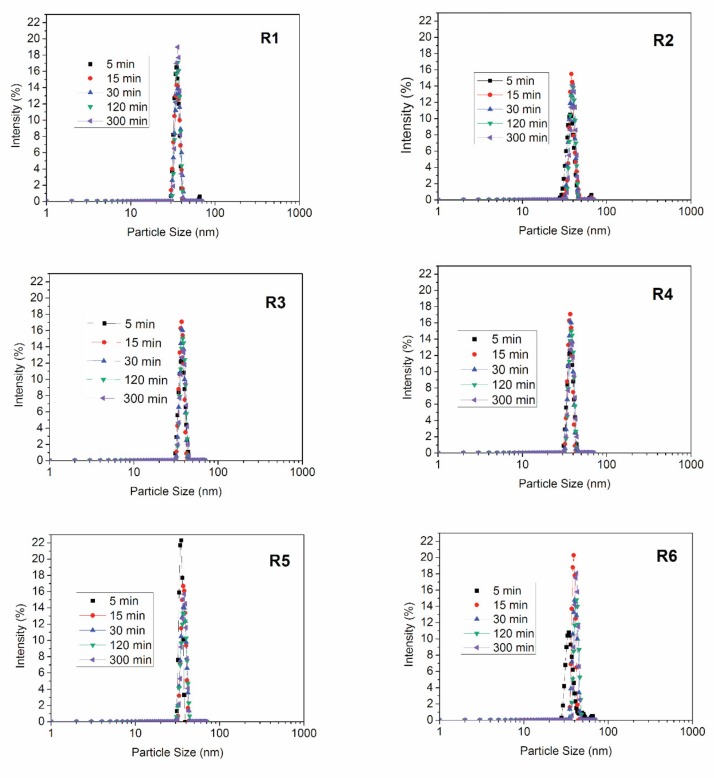
Evolution of particle size distributions for polymerization reactions shown in Table 1.

To prepare the polymer nanoparticles, polymerization reactions were performed in presence and absence of the RAFT agent, as presented in Table 1. In the copolymerization tests, 15 wt % of MMA were replaced by the comonomer. It is very important to report that all prepared emulsions were stable during the course of the reaction and remained stable under storage for at least a couple of months. This information is particularly important because the proposed RAFT miniemulsion copolymerization reactions had not been performed previously and it is not obvious that the latex should be stable at the proposed reaction conditions. Fonseca (2012) [30] and Peixoto (2016) [31] studied the effects of surfactant type and sonication time on the stability of the prepared emulsions and suggested the operation conditions presented in the present manuscript as the most adequate for preparation of the analyzed PMMA nanoparticles. Figure 2 shows the evolution of the particle size distributions for all experiments indicated in Table 1.

**Figure 3:**
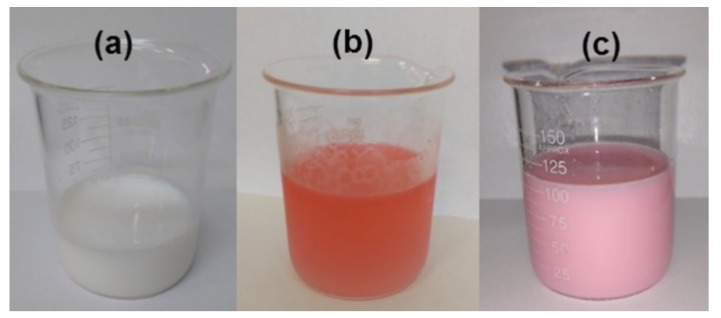
(**a**) MMA emulsion without the addition of RAFT agent; (**b**) MMA emulsion + RAFT agent before sonication; (**c**) MMA emulsion + RAFT agent after sonication.

It was interesting to observe that the color of the emulsion changed when the RAFT agent was used, as shown in Figure 3. When CPBD was not added to the reacting system, the emulsion remained white, but when the red RAFT agent was added, the emulsion became pink after sonication.

**Figure 4:**
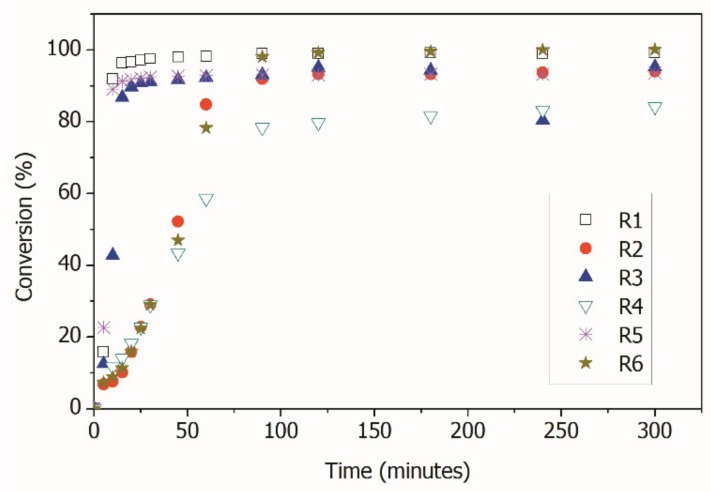
Total Monomer conversions in the distinct reactions.

Figure 4 shows the evolution of monomer conversion in the reactions, indicating, as expected, that the rates of monomer consumption were slower when the RAFT agent was used. This effect was due to the reaction mechanism, which included activation/deactivation reactions of the growing polymer chains, with formation of dormant growing chains [1,8]. Figure 4 also indicates that all reactions seemed to reach very high conversions, with the exception of R4, performed with AA in presence of CPBD. Conventional polymerization reactions reached the final monomer conversions in less than 30 min, while RAFT polymerizations reached final monomer conversions after approximately 90 min.

As shown in Table 2 and Figure 4, all reactions reached conversions that were close to 100% with the exception of R4, when AA was used as the comonomer in the miniemulsion RAFT polymerization. This may be an indication that copolymerization did not occur at significant high rates, as the final conversion was close to the initial MMA content. Therefore, AA may have remained unreacted in the aqueous phase, although additional experimental evidence must be presented to confirm this assumption.

According to [32], the rates of initiator decomposition (K_d_) for AIBN in methyl methacrylate at 50 °C and at 70 °C are, respectively, equal to 97 × 10^−8^ and 3100 × 10^−8^ s^−1^. Based on these data, it is possible to extrapolate that the half life time for AIBN at 80 °C is equal to 1.27 h [16]. Therefore, the low monomer conversion in R4 was not related to the absence of free radicals in the reaction media and probably indicates the low rates of AA incorporation by the growing radicals.

Table 2 also indicates the average molecular weights of the final polymer samples after 5 h of reaction. As one can see, the Mn values of polymers produced without using the RAFT agent were much higher than the Mn values of polymers produced in presence of the RAFT agent. This result could already be expected and is related to the controlled growth mechanism of the RAFT reaction. The theoretical Mn value was calculated using Equations (1) and (2) [33] and inserted into Table 2 for comparison.

(1) $$\mathrm{Mn}=\frac{{\left[M\right]}_{0}}{{\left[\mathrm{RAFT}\right]}_{0}}x\propto {x\mathrm{MM}}_{\mathrm{monomer}}+{\mathrm{MM}}_{\mathrm{RAFT}}$$

where [M]_0_ is the initial molar monomer concentration, [RAFT]_0_ is the initial RAFT agent molar concentration, α is the monomer conversion, MM_monomer_ is the molar mass of the monomer and MM_RAFT_ is the molar mass of the RAFT agent. It is important to highlight that, for R4 and R6, where two different monomers were used, the MM_monomers_ was calculated using Equation (2).

(2) $$\frac{1}{{\mathrm{MM}}_{\mathrm{monomers}}}=\frac{w}{{\mathrm{MM}}_{\mathrm{MMA}}}+\frac{\left(1-w\right)}{{\mathrm{MM}}_{\mathrm{Com}}}$$

where MM_monomers_ is the molar mass of the mixture of monomers, MM_MMA_ is the molar mass of methyl methacrylate and MM_Comc_ is the molar mass of the comonomer and w is the weight fraction of each monomer.

**Figure 5:**
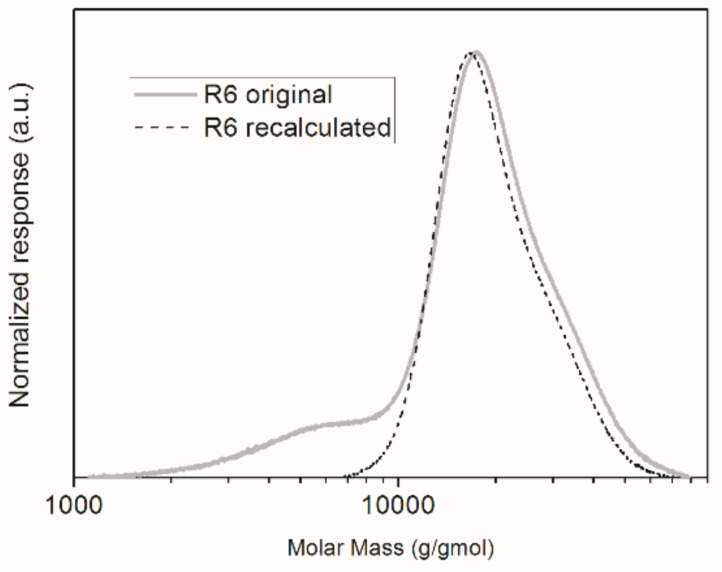
GPC chromatograms of the final polymer product of R6.

As one can notice, with the exception of R6, the obtained Mn values were very close to the theoretical Mn values. However, the difference observed between the experimental number average molar mass and the theoretical Mn value for R6 does not mean that the controlled reaction was unsuccessful. In fact, this may be an indication that methacrylic acid partially dissolved in the aqueous phase and homopolymerized. This might cause the molar mass to be slightly lower than the calculated Mn value because AIBN is not soluble in water; consequently, given the low concentration of monomer and initiator in the aqueous phase, polymerization in the continuous phase tends to form oligomers. In fact, when the original GPC chromatogram for the polymer product of R6 is observed in Figure 5, one can notice that there is a shoulder located in the region of lower molar masses. If this shoulder were disregarded and the calculations repeated, the experimental Mn value would become equal to 1.8 × 10^4^ g/gmol, which is much more similar to the expected Mn value than reported before. Moreover, the PDI decreases from 1.50 to 1.14, indicating that the RAFT reaction proceeded in the disperse phase. This indicates indirectly that a small part of the initial load of methacrylic acid dissolved in water and homopolymerized.

Another interesting GPC result shown in Table 2 is the polydispersity index. In all cases, when the RAFT agent was used, the PDI decreased when compared to the corresponding reactions performed without the RAFT agent, as expected. This indicates that the addition of comonomers did not exert a negative influence on the course of the RAFT reactions; otherwise, the PDI values would have remained high as in the conventional polymerizations. Moreover, the decrease in PDI values indicates that the use of CPBD as a RAFT agent in the proposed reactions can be successful.

**Figure 6:**
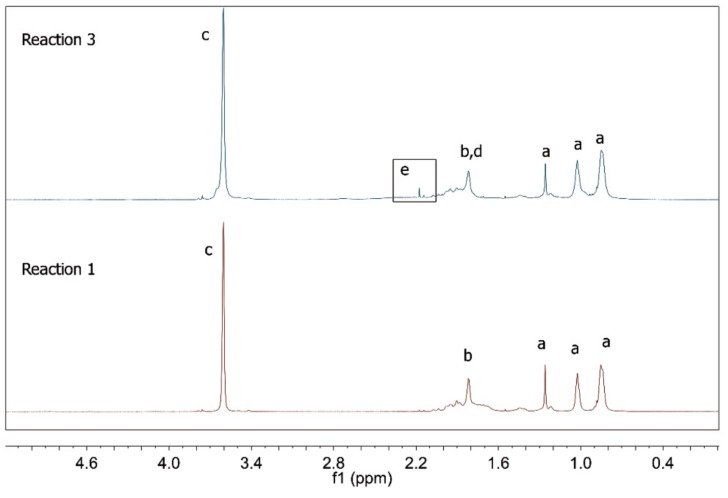
^1^H NMR spectra of polymer products of R1 and R3.

**Figure 7:**
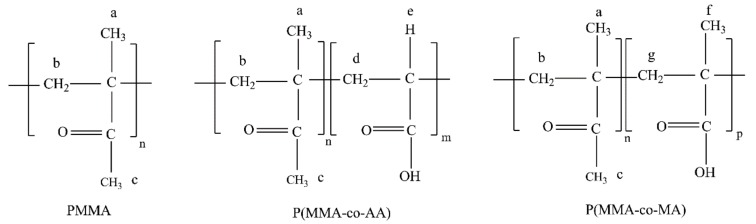
Chemical structure of the (co)polymers and their correspondence to the ^1^H NMR spectra.

According to Table 2, when R1 is compared to R2 and R5 is compared to R6, significant increase of the number average particle size could be observed in presence of the RAFT agent. This was probably caused by the lower rates of the RAFT reactions, as already discussed. Therefore, since miniemulsions are thermodynamically unstable [34], it seems reasonable to believe that, when the polymerization time increased, the mean particle size also increased, indicating that the initial miniemulsion system had not been completely stabilized. However, when R3 and R4 were compared, this particle size difference could not be observed. This may indicate that the acrylic acid dissolved in the aqueous phase and provided additional stabilization of the emulsified particles through homopolymerization in the aqueous media. To confirm this hypothesis, ^1^H NMR analyses were performed and the results for the conventional polymerizations are presented in Figure 6 and Figure 7.

As shown in Figure 6, the characteristic PMMA peaks appear in both spectra [35,36]. However, in R3 a weak signal located at 2.17 ppm indicates the presence of CH protons that are characteristic of poly(acrylic acid) [37]. In fact, when the peak areas obtained for hydrogens “c” and “e” (see Figure 7) are correlated, it is possible to calculate that 6.9% of AA was incorporated. This information reinforces the assumption that acrylic acid did not completely copolymerize with MMA at the analyzed reaction conditions. Similar results were obtained for R2 and R4. It is important to observe that the product of R5 did not dissolve in CDCl_3_ and that the ^1^H NMR analysis of the polymer sample was not possible. However, this information indicates the modification of the chemical structure of the polymer product caused by the addition of methacrylic acid.

**Figure 8:**
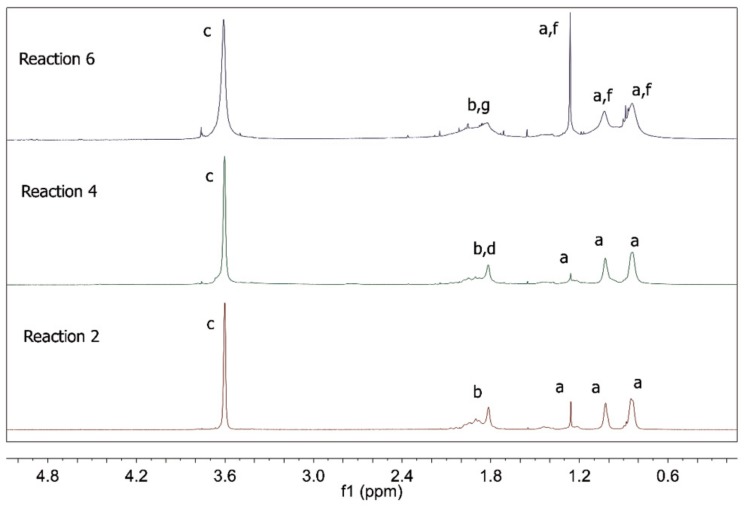
^1^H NMR spectra of polymer products of R2, R4 and R6.

Figure 8 presents the ^1^H NMR spectra of the polymer products of the RAFT reactions. As already discussed for R1 and R3, R2 and R4 provided products with all the characteristic features of PMMA [35,36], indicating once more the low extent of the copolymerization reactions between MMA and AA. Unfortunately, the copolymer composition for R4 could not be calculated as in R3 because hydrogen “e” did not appear in the NMR result for R4. However, when R6 is taken into consideration, it becomes evident that the ^1^H NMR spectrum of the polymer product indicates the insertion of MA into the copolymer structure, given the significant modifications of the characteristic PMMA peaks. It is important to consider, however, that part of the product of R6 did not dissolve in chloroform. Once again, it was not possible to calculate the copolymer compositions for R5 and R6 because MMA and MA structures are very similar, which causes the peaks to overlap.

**Figure 9:**
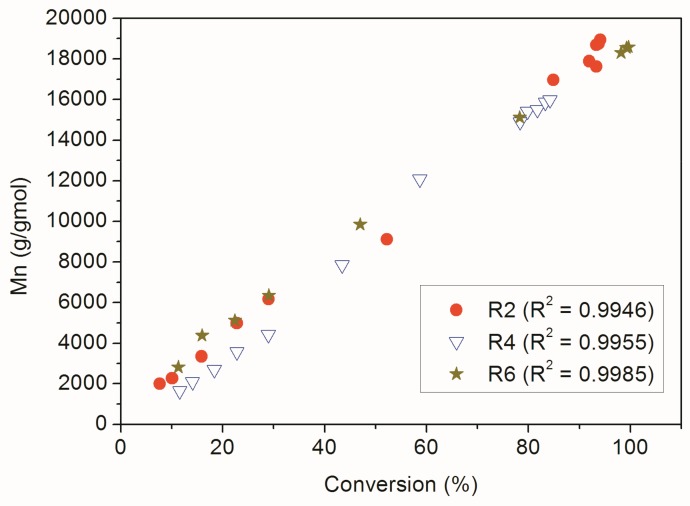
Dynamic evolution of Mn and monomer conversion for RAFT reactions.

The relative increase of the solubility of MMA/MA copolymers in chloroform can possibly be related to the lower average molar masses of the polymer materials prepared in presence of the RAFT agent. However, this could also be related to thermodynamic constraints, given the partial solubility of MA in water (89 g/L), and modification of reactivity ratios in the RAFT system [38]. For instance, it must be acknowledged that RAFT agent may react more efficiently with methacrylate monomers than with acrylate monomers [39]. However, Figure 9 shows that the RAFT agent was able to control the chain growth appropriately in all analyzed polymerization reactions, since the number average molar mass of the polymers increased linearly with the monomer conversion in all cases.

**Figure 10:**
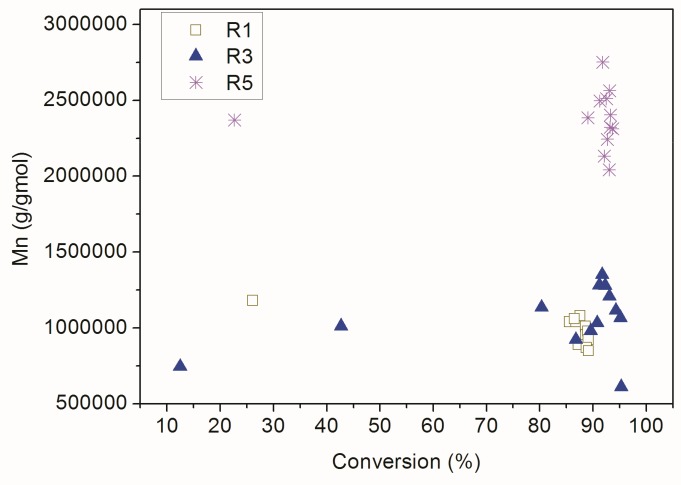
Dynamic evolution of Mn and monomer conversion in the conventional reactions.

On the other hand, for the conventional polymerization reactions the linear dependence between Mn and monomer conversion was not observed, as one can see in Figure 10 and might already expect. When the RAFT agent was not added, the polymer chains grew extremely quickly and the number average molar mass of the polymer material in the first instants of the reaction was very similar to the final number average molar mass of the product. Once again, these results indicate that 2-cyano-2-propyl-dithiobenzoate was able to control the proposed RAFT reactions. Additionally, the similar Mn profiles obtained in R1 and R3 suggest the low incorporation of AA into the copolymer chains.

Contact angle measurements were also performed to determine the hydrophilicity of the produced polymers and the results are presented in Table 3. According to Table 3, the addition of the comonomers enabled the increase of the polymer affinity with water, as expressive reduction of the contact angles was observed in presence of the comonomers. Particularly, addition of the RAFT agent did not influence the hydrophilicity of the polymer products significantly, as one might expect, given the very low amounts of CPBD in the polymer chains. It is also worth noticing that the contact angles of the polymer product prepared in presence of AA were much lower than the ones obtained for PMMA homopolymers, indicating that the low amounts of AA incorporated into the chains were sufficient to modify the degree of hydrophilicity of the final materials. Besides, when the contact angles of polymer products obtained in R3 and R4 are compared to each other, it becomes possible to conclude that incorporation of AA in the copolymer chains was even smaller in presence of the RAFT agent. Finally, Table 3 confirms the much more efficient incorporation of MA into the final copolymer chains, when compared to AA.

Results obtained after cell uptake analyses are presented in Table 4 and can be divided into two groups. The first group, comprising Samples A–C, was subjected to the bioconjugation reaction with the maleimide-PEG-NHS spacer, meaning that the biomolecule was chemically bonded to the polymer particles [18,23,27]. It is important to highlight that the RAFT agent is responsible for obtaining polymers with narrow molar mass distributions, but, in this work, it also participated in the bioconjugation reaction. The second group, comprising Samples D–F, was prepared by mixing the latex to the BSA solution, meaning that BSA was adsorbed onto the nanoparticle surface [40]. It is important to highlight that the only difference between the procedures used to prepare the samples in the different groups was the addition of maleimide-PEG-NHS.

To quantify the amount of BSA adsorbed or bounded to the polymer particles, Bradford protein assays were conducted with the supernatant of all samples collected, before labelling with rhodamine B. The bioconjugation efficiencies determined for each sample are presented in Table 5.

According to Table 5, high bioconjugation efficiencies could be obtained through adsorption and in absence of the comonomers, although efficiencies were seemingly higher in presence of the maleimide-PEG-NHS spacer. The results presented in Table 5 indicate that the bioconjugation efficiencies were equal to at least 85% in presence of the spacer molecule and equal to at least 70% in absence of the spacer molecule. Apparently, the addition of comonomers exerted little influence on the bioconjugation efficiencies, although higher bioconjugation efficiencies could be assigned to the higher hydrophobicity levels, which can be related to the intrinsic BSA characteristics. Based on Table 5, the use of the maleimide-PEG-NHS spacer should not be regarded as extremely necessary in this case, making bioconjugation simpler.

**Figure 11:**
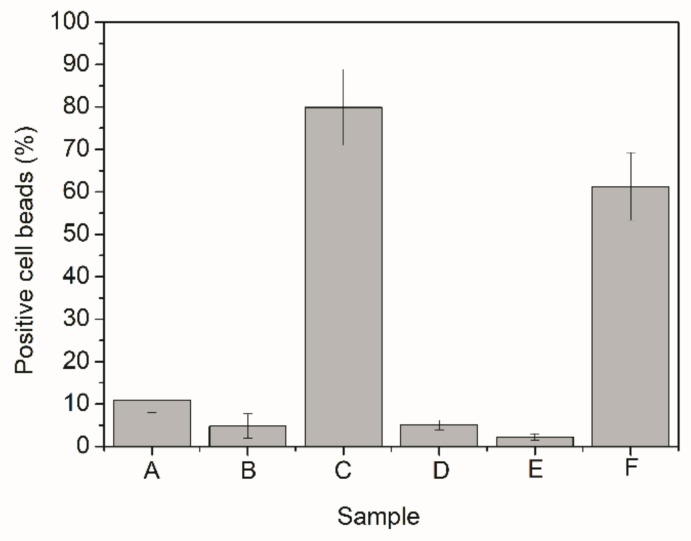
Cellular uptake of polymer particles bioconjugated with BSA.

To analyze the cell uptake results, presented in Figure 11, it is important to group the samples in different ways. Confronting first the results obtained for samples that used the spacer molecule with results obtained for samples that were only mixed to BSA, as in Samples A and D, it becomes possible to observe that the cell uptake increased when maleimide-PEG-NHS was added. This increase could also be noticed for the other samples (Samples B and E; and Samples C and F). These results might be related to the higher amounts of BSA on the particle surface and/or to the stronger links between the polymer material and the protein in presence of the maleimide-PEG-NHS spacer. Despite that, the results obtained in both cases were not much different, encouraging the use of simple adsorption for bioconjugation.

On the other hand, comparing cell uptake results obtained for Samples A–C, it can be observed that the polymer particles that led to the best performance were the ones prepared in presence of methacrylic acid, followed by the PMMA homopolymer and finally the copolymer prepared in presence of acrylic acid. The very same behavior was observed when Samples D–F were compared to each other. The results can be regarded as very consistent and indicate that macrophages preferably internalize the product of R6, which was the material with the highest hydrophilicity (or lowest contact angle). This seems to suggest that increasing the hydrophilicity of the polymers improves the performance of the system and may also explain why Sample A, which presented the highest bioconjugation efficiency, does not show highest cell uptake [4,5,6]. However, the lowest cell uptakes were obtained for the product of R4, which presented intermediate contact angle values, clearly indicating that particle hydrophilicity may not be the only influential factor in the problem. Particularly, the obtained cell uptake performance of the product prepared in presence of methacrylic acid suggests that the particle composition (particularly the composition of the particle surfaces) can exert a very significant effect on the final cell uptake results, encouraging the development of more extensive cell uptake analyses for other copolymer materials.

## 4. Conclusions

Miniemulsion polymerizations of methyl methacrylate (MMA) were performed in presence of acrylic acid (AA) and methacrylic acid (MA), using 2-cyano-2-propyl-benzodithioate (CPBD) as RAFT agent. As shown through different analyses, the RAFT agent allowed for successful free radical controlled MMA copolymerizations, although incorporation of AA into the polymer chains was limited to very low extents. In all cases, the copolymerization reactions allowed for increase of the hydrophilicity levels of the polymer materials. Particularly, the contact angles of the copolymers prepared in presence of MA were lower than the contact angles of the copolymers prepared in presence of AA, which were in turn lower than the contact angles of the PMMA homopolymers.

The obtained results also indicate that the use of the maleimide-PEG-NHS spacer allowed for more efficient bioconjugation of BSA onto the polymer particles, although bioconjugation through adsorption also allowed for high bioconjugation efficiencies. However, particles prepared in presence of the polar comonomers caused slight decrease of the BSA bioconjugation efficiencies, because of the hydrophobic nature of BSA. Despite that, cellular uptake results were much higher for particles prepared in presence of MA, indicating the tremendous effect exerted by the copolymer composition on the internalization of copolymer particles by macrophages.

Further studies involving RAFT polymerization, drug encapsulation and bioconjugation may allow the development of targeted drug delivery systems in the future.

## Figures and Tables

**Table 1:** Comonomer compositions in the polymerization reactions using methyl methacrylate as the main monomer.

Reaction	Comonomer *	RAFT Agent
R1	-	No
R2	-	Yes
R3	Acrylic acid	No
R4	Acrylic acid	Yes
R5	Methacrylic acid	No
R6	Methacrylic acid	Yes

* When other monomers were added, 15% of MMA was replaced by the respective comonomer.

**Table 2:** Conversion (%), number-average molar mass (Mn), polydispersity (PDI) and after particle size of final polymer products obtained after 300 min of reaction.

Reaction	Conversion (%)	Theoretical Mn (g/gmol)	Mn (g/gmol)	PDI	Mean Particle Size (nm)
R1	99	-	1.13 × 10^6^	1.93	72.6 ± 0.4
R2	94	1.98 × 10^4^	1.89 × 10^4^	1.13	131.1± 0.7
R3	95	-	6.11 × 10^6^	3.33	103.6 ± 1.4
R4	84	1.67 × 10^4^	1.60 × 10^4^	1.57	104.6 ± 1.5
R5	94	-	2.31 × 10^6^	1.62	94.1 ± 0.8
R6	100	2.00 × 10^4^	1.34 × 10^4^	1.50	166.1 ± 0.3

**Table 3:** Contact Angle of the final polymer products.

Reaction	Contact Angle
R1	77.2° ± 4.4
R2	74.6° ± 1.1
R3	39.4° ± 2.7
R4	52.8° ± 1.6
R5	39.3° ± 1.2
R6	35.2° ± 2.1

**Table 4:** Description of the samples submitted to cell uptake analyses.

Sample	Description
A	R2 + maleimide-PEG-NHS + BSA
B	R4 + maleimide-PEG-NHS + BSA
C	R6 + maleimide-PEG-NHS + BSA
D	R2 + BSA
E	R4 + BSA
F	R6 + BSA

**Table 5:** Description of the samples submitted to cell uptake analyses.

Sample	Bioconjugation Efficiency
A	93%
B	85%
C	85%
D ^1^	77%
E, F ^1^	70%

^1^ extracted from [40].
